# The Diagnostic Challenge of Eosinophilic Granulomatosis With Polyangiitis Presenting as Acute Eosinophilic Myocarditis: Case Report and Literature Review

**DOI:** 10.3389/fcvm.2022.913724

**Published:** 2022-07-07

**Authors:** Hiroyuki Yamamoto, Katsuya Hashimoto, Yoshihiko Ikeda, Jun Isogai, Toru Hashimoto

**Affiliations:** ^1^Department of Cardiovascular Medicine, Narita-Tomisato Tokushukai Hospital, Chiba, Japan; ^2^Department of Pathology, National Cerebral and Cardiovascular Center, Suita, Japan; ^3^Department of Radiology, Asahi General Hospital, Asahi, Japan

**Keywords:** EGPA, acute EM, hypereosinophilia, CMR, EMB, corticosteroid treatment

## Abstract

Eosinophilic granulomatosis with polyangiitis (EGPA) is a systemic vasculitis involving small-to-medium-sized vessels characterized by asthma, vasculitis, and peripheral eosinophilia. EGPA-associated eosinophilic myocarditis (EM) occurs rarely, yet can be fatal if left untreated. Moreover, the accurate diagnosis of EGPA-associated EM without vasculitis is exceptionally difficult because of the overlapping features with EM of other causes. We report a case of probable EGPA with subclinical neurological involvement that presented with acute EM. The constellation of peripheral eosinophilia, left ventricular dysfunction, and normal epicardial coronary arteries raised suspicion of acute EM, which was confirmed by cardiac magnetic resonance (CMR) investigation and endomyocardial biopsy (EMB). Prompt systemic administration of corticosteroids completely restored and normalized myocardial structure and function. Although the patient's history suggested the presumed hypersensitivity myocarditis, EMB revealed EM without vasculitis, not hypersensitivity, leading to a tentative diagnosis of idiopathic hypereosinophilic syndrome. Interestingly, the characteristic findings of vasculitis on CMR imaging strongly suggested EGPA-associated EM. Although the patient had no clinical neurological manifestations, a nerve conduction study confirmed mononeuritis multiplex, leading to the final diagnosis of probable EGPA. Therefore, this case highlights the diagnostic challenge associated with EGPA and the diagnostic synergy of CMR and EMB for an exploratory diagnosis of EGPA-associated EM.

## Introduction

Eosinophilic granulomatosis with polyangiitis (EGPA), previously known as Churg-Strauss syndrome, is a multisystem disorder characterized by necrotizing vasculitis of small-to-medium-sized vessels, with the coexistence of asthma, rhinosinusitis, and marked peripheral eosinophilia (1). EGPA-associated eosinophilic myocarditis (EM) is rare but can be fatal (1, 2). Because EM has multiple etiologies with overlapping clinical and biological features, a definitive diagnosis of EGPA-associated EM remains challenging in the absence of clinical manifestations of vasculitis. Therefore, a promising strategy to accurately diagnose EGPA-associated EM needs to be formulated.

## Case Description

**Figure 1:**
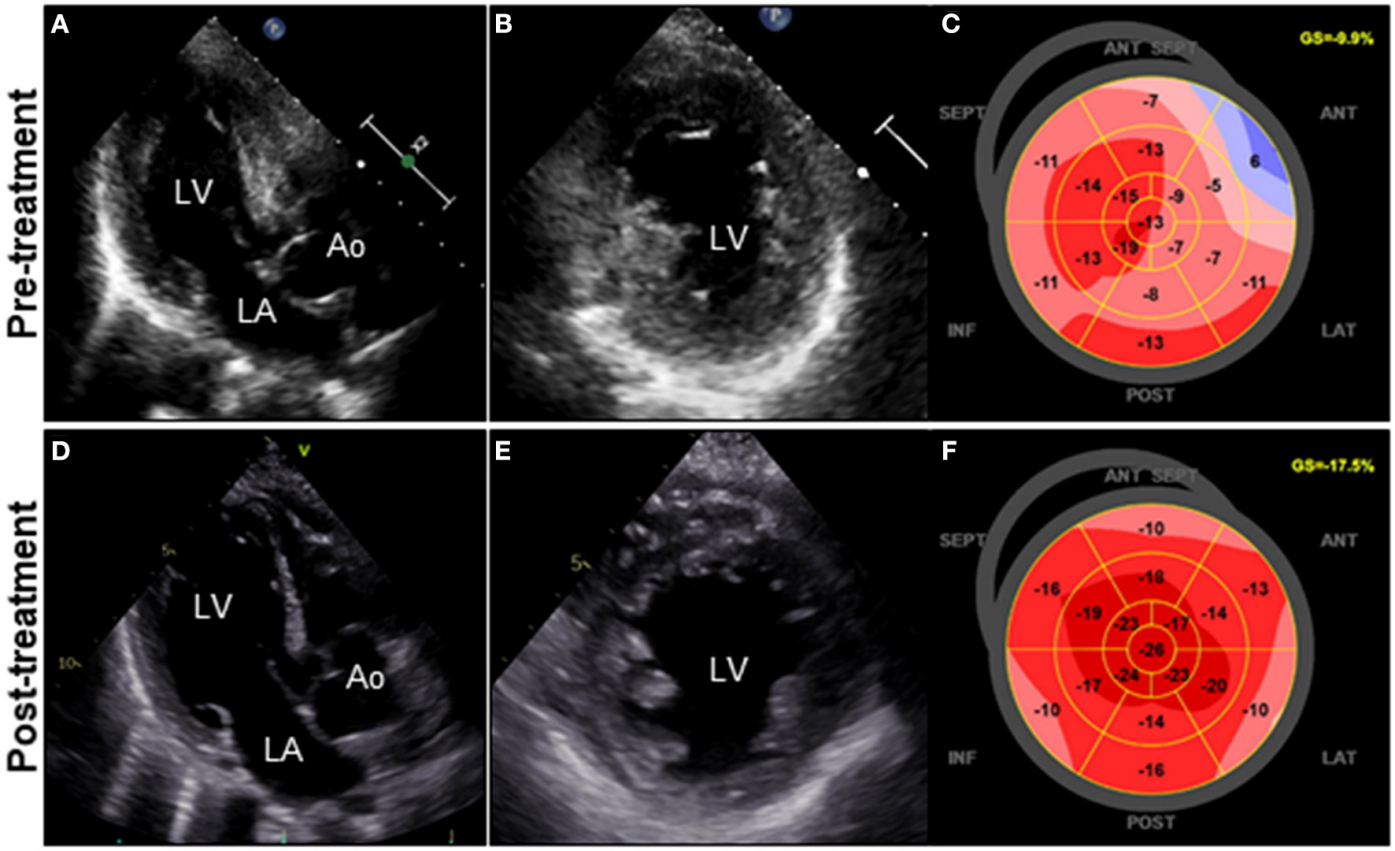
Clinical effects of corticosteroid treatment on TTE. TTE on admission reveals diffuse and symmetrical LV wall thickening (11 mm), decreased cavity size, reduced ventricular function, and GLS values of the LV (LVDd, 44 mm; LVEF, 43%; and GLS, −9.9%; respectively) **(A–C)**. Follow-up TTE on day 21 after corticosteroid therapy reveals a significant decrease in LV wall thickness (8 mm) with concomitant improvement in cavity size, ventricular function, and GLS values of the LV (LVDd, 48 mm; LVEF, 50%; and GLS, −17.5%; respectively) **(D–F)**. Ao, aorta; GLS, global longitudinal strain; LA, left atrium; LV, left ventricle; LVDd, left ventricular end-diastolic diameter; LVEF, left ventricular ejection fraction; TTE, transthoracic echocardiography.

**Figure 2:**
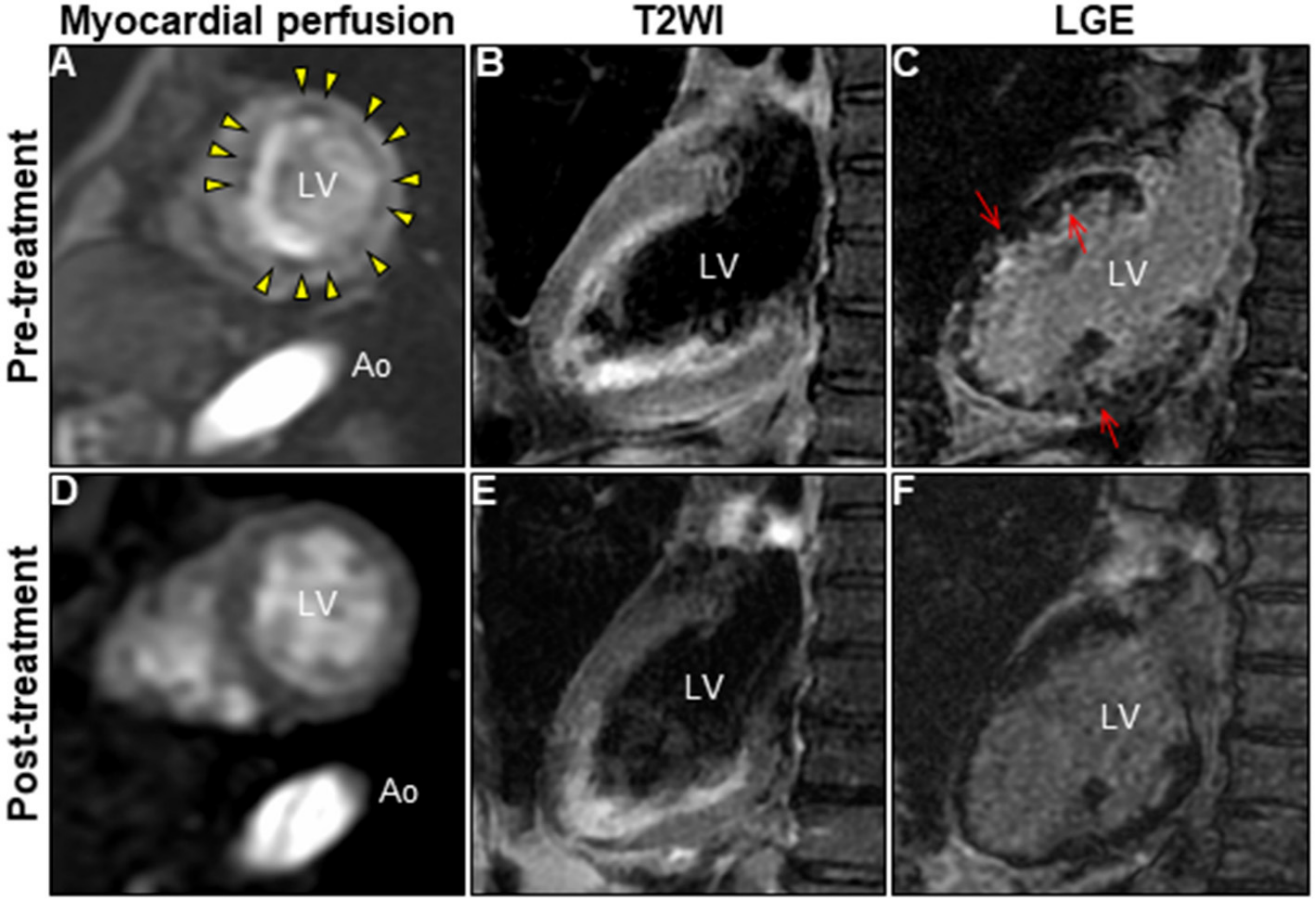
Changes in CMR findings in patients following corticosteroid treatment. CMR findings before **(A–C)** and after corticosteroid treatment **(D–F)**. Myocardial first-pass perfusion imaging at rest **(A,D)**, T2WI of the 2-chamber view **(B,E)**, and LGE image **(C,F)**. Ao, aorta; CMR, cardiac magnetic resonance; LGE, late gadolinium enhancement; LV, left ventricle; T2WI, T2-weighted image.

**Figure 3:**
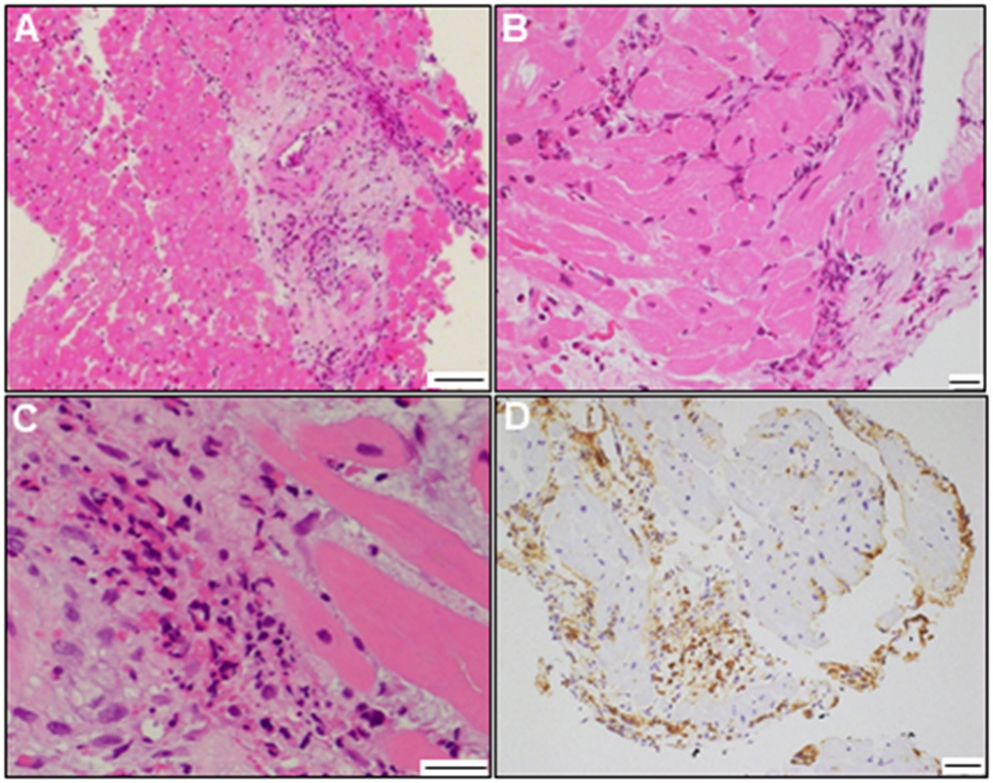
Endomyocardial biopsy findings. Photomicrograph with hematoxylin and eosin staining **(A–C)** (bars: A, 100 μm; B and C, 20 μm). Photomicrograph showing immunostaining against the major basic protein **(D)** (bar: 50 μm).

A 72-year-old woman was admitted to our hospital with worsening chest pain. She had a history of asthma and was treated with a fluticasone furoate/vilanterol (inhaler), theophylline (200 mg/day), ambroxol hydrochloride capsules (45 mg/day), montelukast (10 mg/day), and mequitazine (6 mg/day). Her treatment was aided with short-term use of oral prednisolone as required. The patient's general practitioner switched her from regular branded medications to generic medications 1 month prior to her admission. A week before admission, she experienced chest pain, characterized by chest tightness on exertion that disappeared on rest. Her symptoms worsened and were accompanied by dyspnea on effort (New York Heart Association class II–III). Her vital signs were as follows: blood pressure 118/73 mmHg, heart rate 87 beats/min, temperature 37.3°C, and oxygen saturation 97%. The physical and neurological examination results and chest radiographs were unremarkable. The electrocardiogram (ECG) showed pathologic Q waves in V2 to V3, and negative QRS complexes including rS morphology in the inferior leads, diagnosed as a left anterior fascicular block (Supplementary Figure 1A). The laboratory testing revealed a significant eosinophilia with an eosinophil percentage of 67.6% (normal <6%) and an absolute eosinophil count (AEC) of 10,410/μL (normal <500/μL), elevated brain natriuretic peptide level of 536 pg/mL (normal <18.4 pg/mL), and elevated cardiac biomarkers levels as follows: creatine kinase, 473 U/L (reference: 41–153 U/L); CK-MB, 39 U/L (normal <25 U/L); aspartate aminotransferase, 72 U/L (reference: 13–30 U/L); lactate dehydrogenase, 613 U/L (reference: 124–222 U/L); and high sensitivity cardiac troponin I, 47,875.3 pg/mL (normal <26.2 pg/mL). The levels of the inflammatory markers were also elevated—the C-reactive protein was 0.65 mg/dL (normal <0.3 mg/dL) and the erythrocyte sedimentation rate was >110 mm/h (reference: 3–15 mm/h). The results of renal function and urinalysis were normal. Further laboratory studies revealed elevated levels of serum IgE at 1,840 IU/mL (normal <173 IU/mL) and rheumatoid factor at 204 IU/mL (normal <15 IU/mL). In addition, the levels of the Th2 cytokines-related interleukins (IL) were also raised—IL-4 was 7.7 pg/mL (normal <3.9 pg/mL) and IL-5 was 30 pg/mL (normal <3.9 pg/mL). Anti-neutrophil cytoplasmic antibodies (ANCA) were not detected. Echocardiography revealed a mildly thickened myocardium and significant left ventricular (LV) systolic dysfunction with an ejection fraction of 43%. In addition, speckle-tracking echocardiography showed a reduced baseline global longitudinal strain of −9.9% (Figures 1A–C and Supplementary Video 1). Accordingly, we made a tentative diagnosis of acute coronary syndrome (ACS). An emergency coronary angiography was performed after pre-treatment with methylprednisolone (250 mg) that was administered to prevent allergic contrast reactions for the patient with asthma. The angiogram revealed normal epicardial coronary arteries. Therefore, acute EM was suspected, and cardiac magnetic resonance (CMR) was performed to assess the myocardial tissue (Figures 2A–C and Supplementary Video 2). Myocardial first-pass perfusion (FPP) imaging showed patchy and circumferential subendocardial perfusion defects (arrowheads), suggesting microvascular disorders. CMR also showed subendocardial late gadolinium enhancement (LGE) as multiple and lobulated high-signal intensity spots (arrows), which suggested vasculitis. The T2-weighted image showed a transmural high-intensity signal throughout the myocardium, corresponding to myocardial edema. Acute myocarditis was diagnosed based on the Lake–Louise criteria. These findings were consistent with acute EM. Simultaneously, an exhaustive diagnostic workup for hypereosinophilia was performed. Its differential diagnoses include hypersensitivity myocarditis (HSM), EGPA, parasitic infections, hematologic malignancies, and lymphocytic/idiopathic hypereosinophilic syndrome (HES). Considering the patient's recent medical history, HSM was initially suspected as the cause of EM. The generic drugs being administered to the patient were discontinued after admission. A subsequent endomyocardial biopsy (EMB) was performed, which demonstrated marked extravascular eosinophilic infiltrates without granulomatous and fibrinoid necrotizing vasculitis (Figure 3A). Numerous eosinophilic infiltrations, with degranulated eosinophils admixed with lymphocytes and myocyte necrosis, were observed in the myocardial interstitium that extended to the endocardium (Figures 3B,C). Moderate endocardial thickening and perivascular interstitial fibrosis were observed (data not shown). Immunostaining was performed to identify the major basic proteins revealed extensive staining in the endocardium and myocardial interstitium (Figure 3D). These findings led to the final diagnosis of acute EM. Subsequently, the patient was treated with intravenous methylprednisolone (1 g/day for 3 days), followed by oral prednisolone (1 mg/kg/day). The clinical response to steroid treatment was remarkable, with significant recovery of LV dysfunction, hypereosinophilia, and elevated cardiac enzyme levels within 21 days of steroid treatment (Figures 1D–F and Supplementary Video 3). Follow-up ECG showed resolution of all abnormal findings recognized during the initial ECG (Supplementary Figure 1B). On day 33, the patient was discharged after administration of prednisolone (15 mg/day), with a gradual tapering of the doses. On day 56, the patient remained asymptomatic, with fully recovered cardiac function observed on echocardiography (Supplementary Figure 2 and Supplementary Video 4). Moreover, the abnormal findings of the CMR resolved completely (Figures 2D–F and Supplementary Video 5). On day 75, prednisolone was tapered and finally discontinued in the outpatient clinic. However, her asthma precipitated again 2 weeks later, which was concurrent with an eosinophilia count of 1,512/μL. Since a thorough diagnostic workup for hypereosinophilia was negative, idiopathic HES was also considered. Although the patient had no clinical neurological manifestations, her CMR findings were suggestive of vasculitis, which encouraged us to perform a nerve conduction study that revealed mononeuritis multiplex. Eventually, as per the diagnostic criteria of the American College of Rheumatology (ACR) for EGPA, the patient met four of the six items (asthma, eosinophilia >10%, mononeuritis multiplex, and extravascular eosinophilia). However, a histological diagnosis of vasculitis could not be performed because the patient refused nerve biopsies. Therefore, the final diagnosis of probable EGPA was made. The patient was restarted on prednisolone treatment (15 mg/day). At the 1-year follow-up, the patient remained clinically stable, with prednisolone tapered to 4 mg/day. As a supplement, we have presented a timeline for the case presentation (Supplementary Figure 3).

## Discussion

EGPA is classified among ANCA-positive vasculitides, and its underlying pathological mechanism remains poorly understood. The widely accepted ACR criteria for EGPA include asthma, eosinophilia (>10% in the differential count), neuropathy, non-fixed pulmonary infiltrates, paranasal sinus abnormalities, and extravascular eosinophils. According to a study, the ACR criteria for the classification of vasculitis as EGPA yielded a sensitivity of 85.0% and specificity of 99.7%, when four or more of the aforementioned conditions were applicable (3). EGPA is commonly diagnosed at ~40 years of age and exhibits no gender predominance. Its reported prevalence is 10.7–18 per million in Europe and the United States (4, 5). EGPA progresses through three chronological phases: the prodromal phase with the occurrence of asthma and allergic manifestations; the eosinophilic phase, which is characterized by eosinophilic infiltration of the organs involving the lungs and the myocardium; and the vasculitic phase, which is characterized by organ damage due to vasculitis in small-to-medium-sized vessels in the skin, peripheral nerves, and kidneys. These three phases may overlap. Generally, EGPA has an excellent prognosis, with a 5-year survival rate of 97% (6). Cardiovascular involvement rarely occurs (about 16% of organ involvements), but is the leading cause of death in about 50% of cases (7). Although data on cardiac involvement in patients with EGPA are available in the literature, the number of reported cases of EGPA-associated EM is small (2, 8). Therefore, we conducted an updated systematic review of case reports to investigate the characteristics of EGPA-associated EM (Table 1). The literature search was performed using PubMed databases documented from 1983 to 2021, with the search restricted to studies published in English. We used the following MeSH terms: “eosinophilic granulomatosis with polyangiitis” or “Churg–Strauss syndrome” and “case report” and “myocarditis.” Moreover, we selected cases of EGPA-associated EM confirmed by EMB and/or CMR findings. Finally, 38 cases were included (9–46). The mean age of the participants included was 47 years with no gender predominance. The main extracardiac involvement was asthma, followed by peripheral neuropathy and constitutional symptoms (94.7, 52.6, and 23.7%, respectively). At presentation, the patients were commonly diagnosed with cardiac insufficiency (51.6%), ACS (21.1%), and cardiogenic shock (13.2%). As in our case, patients with EGPA-associated EM had a high rate of ANCA-negativity and presented with marked peripheral eosinophilia. Presence of LV dysfunction (66%) and pericardial effusion (47%) were frequently observed. A higher five-factor score was indicative of poor prognosis in 39% of the patients. Considering the number of cases with a poor clinical course, including sudden cardiac death (21%) and incomplete normalized cardiac function despite intensive treatment (26%), early recognition and treatment of EGPA-associated EM is required.

**Table 1:** Cases of EGPA-associated EM.

**Author**	**Case**	**Age/sex**	**Clinical features (Extracardiac organ involvement)**	**Diagnosis at presentation**	*Revised 2021 FFS	**Laboratory findings**		**Cardiac Imaging**		**Myocardial histopathology**	**LGE pattern on CMR**	**Immunosuppressive treatment**	**Outcome**
						**AEC (%) [/mm^**3**^]**	**ANCA status**	**LVEF [%]**	**PE**				
Lie (9)	1	39/M	BA	SCD	na	na	na	na	na	NEM, Coronaritis	Not performed	Not performed	Died
Terasaki (10)	2	43/F	BA, CS, PNS	CI	2	16,285 (66%)	Present	Reduced	Present	EM	Not performed	GCs	Worsened
Ramakrishna (11)	3	34/F	BA, CS, ENT, PNS, Skin	Right ventricular thrombus, PN	0	3,660 (23%)	Absent	30–35	Absent	NEM, Granuloma	Not performed	pGCs/GCs	Improved
Hayashi (12)	4	26/F	BA, CS, ENT, Lung	CI	1	3,300 (29%)	Absent	19	Present	NEM	Not performed	pGCs/GCs	SCD
Schoppet (13)	5	50/F	BA, PNS	CI	2	na (39%)	na	30	Present	EM, Granuloma	Not performed	GCs/AZA	Partial cardiac recovery
Shanks (14)	6	51/F	BA, PNS, Skin	CS	2	5,600 (60%)	Absent	25	Present	EM	Not performed	GCs	Improved
Petersen (15)	7	53/F	BA, Lung	ACS	1	2,880 (60%)	Absent	na	Absent	Not performed	Diffuse SEndo	GCs	na
Ferrari (16)	8	25/M	None	CS	2	na (50%)	na	<30	Present	EM, Granuloma	Diffuse midwall	GCs/CYC	Partial cardiac recovery
Hervier (17)	9	42/na	BA, ENT	Abdominal pain	0	9,700 (60%)	Absent	na	Absent	NEM, Vasculitis	Not performed	Not performed	SCD due to VF
Setoguchi (18)	10	60/M	BA, PNS	ACS	2	Elevated	Absent	Preserved	Present	NEM, Vasculitis	Not performed	Not performed	SCD
Zardkoohi (19)	11	71/M	BA, CS, ENT, Lung, PNS	CS, ACS	2	1,850 (20%)	Absent	15	Absent	EM, Mural thrombus	Not performed	pGCs/GCs/MTX	Improved
Courand (20)	12	22/M	BA, CS, ENT, GI, Kidney, Lung	CS	2	7,700 (51%)	Absent	30	Present	Lymphocytic Myocarditis	Diffuse SEndo, patchy midwall	pGCs/GCs/pCYC/AZA	Improved
Levine (21)	13	85/F	BA, Joint, Muscle, PNS, Skin	Stroke	2	12,818 (58%)	Absent	57	Absent	EM	Partial SEndo	pGCs/GCs	SCD
McAleavey (22)	14	55/M	BA, ENT, Joint, Skin	Systemic vasculitis	0	15,750 (na)	Absent	Preserved	Present	Not performed	Partial SEndo	pGCs/GCs/CYC	Improved
Correia (23)	15	22/F	BA, CS, ENT, Lung, PNS	ACS	0	3,830 (na)	Absent	Preserved	Present	EM	Not performed	pGCs/GCs/CYC	Improved
Hara (24)	16	67/F	BA, PNS, Skin	CI, PN	2	10,450 (68%)	na	30	Absent	Not performed	SEndo	GCs/CYC	Partial cardiac recovery
Załeska (25)	17	55/M	BA, ENT, Lung	CI	1	3,880 (31%)	Absent	29	Present	Not performed	Diffuse SEndo, patchy midwall	GCs/CYC	Partial cardiac recovery
Bouiller (26)	18	63/F	BA, ENT	Chest Pain and dyspnea	0	3,480 (25%)	Absent	17	Present	EM, Mural thrombus	Patchy midwall and SEpi	pGCs/GCs/pCYC/AZA	Improved
Bouabdallaoui (27)	19	21/M	BA, PNS	CS	2	4,470 (34%)	Absent	15	Absent	Not performed	Patchy SEndo, ICTh	pGCs/GCs	Partial cardiac recovery
Hase (28)	20	50/M	BA, ENT, Joint, Muscle, PNS, Skin	ACS	0	11,432 (55%)	Absent	30–40	Absent	EM	No LGE	GCs	No change
Beck (29)	21	75/F	BA, ENT	ACS	1	Elevated	Present	Preserved	Absent	EM	Diffuse SEndo, ICTh	GCs	Died
Ammirati (30)	22	25/M	ENT	CS	1	na (64%)	na	15	Absent	NEM	Patchy SEndo	pGCs/GCs/MTX	Partial cardiac recovery
Glaveckaite (31)	23	41/F	BA, ENT, PNS	Cardiac tamponade, PE	0	Elevated	na	<45%	Present	Not performed	Diffuse SEndo, ICTh	GCs/AZA	Improved
Bluett (32)	24	28/M	BA, CS, Lung	Perimyocarditis	1	3,400 (25%)	Absent	25	Absent	EM	Patchy SEndo	pGCs/GCs/Imanitib	Partial cardiac recovery
Plenzig (33)	25	51/M	BA, CS	SCD	2	na	na	na	na	EM, Granuloma	Not performed	na	Died
Dalia (34)	26	19/M	BA, ENT, Lung, PNS	AF with RVR	0	12,960 (45%)	Absent	55	Present	Not performed	Patchy midwall	pGCs/GCs/pCYC	Improved
Miyazaki (35)	27	60/M	BA, PNS	CI	2	15,310 (61%)	Absent	40	Absent	EM	Diffuse SEndo with Foci	pGCs/GCs/AZA	Improved
Ali (36)	28	65/F	BA, ENT, PNS, Skin	CI	2	23,000 (na)	Absent	28	Absent	EM	Patchy midwall	pGCs/GCs/AZA	Partial cardiac recovery
Ferreira (37)	29	65/F	BA, ENT, Muscle, PNS, Skin	Systemic vasculitis	1	11,280 (60%)	Absent	32	Present	Not performed	Patchy SEndo with Foci	pGCs/GCs/CYC	Improved
Dey (38)	30	60/F	BA, CS, ENT, Kidney, PNS, Skin	ACS	1	17,000 (69%)	Present	Preserved	Present	Not performed	Patchy midwall and Tm	pGCs/GCs/CYC/AZA	Improved
Chaudhry (39)	31	68/F	BA, ENT, GI, Kidney	CI, ischemic colitis	2	8,463 (39%)	Absent	20–25	Absent	NEM	Not performed	pGCs/GCs	Died
Gill (40)	32	44/F	BA, ENT, Lung	ACS	0	7,230 (42%)	na	na	Present	Not performed	Patchy SEndo	pGCs/GCs/CYC/AZA	Improved
Lopes (41)	33	22/M	BA, ENT, Lung, PNS	CI	1	11,700 (48%)	Absent	<30%	Absent	EM	Diffuse SEndo	pGCs/GCs/pCYC	Partial cardiac recovery
Civelli (42)	34	62/M	BA, CNS, GI, Kidney, Skin	CI	2	13,046 (46%)	Absent	18	Present	EM	Diffuse SEndo	pGCs/GCs/CYC	Improved
Colantuono (43)	35	19/M	BA, GI, PNS, Skin	Colitis	1	13,470 (na)	Absent	40	Absent	EM	Diffuse SEndo with Foci	pGCs/GCs/Benralizumab	Improved
Higashitani (44)	36	46/F	BA, Lung, Muscle, PNS	Muscle weakness, CI	2	3,250 (na)	Absent	41	Present	EM	Patchy midwall	pGCs/GCs/RTX/MPZ	Partial cardiac recovery
Kurihara (45)	37	66/F	BA, PNS	CI	2	30,609 (86%)	Absent	40	Absent	EM	Not performed	GCs	na
Inaba (46)	38	61/M	BA, CNS, CS, ENT, Skin	Systemic vasculitis	0	7,788 (45%)	Present	Preserved	Absent	Not performed	Foci	pGCs/GCs/CYC/IVIG	Improved

*EGPA, eosinophilic granulomatosis with polyangiitis; BA, bronchial asthma; CS, constitutional symptoms including fever, malaise, and weight loss; PNS, peripheral nervous system; ENT, ear-nose-throat; GI, gastrointestinal; CNS, central nervous system; SCD, sudden cardiac death; CI, cardiac insufficiency; PN, peripheral neuropathy; CS, cardiogenic shock; ACS, acute coronary syndrome; PE, pericardial effusion; AF with RVR, atrial fibrillation with rapid ventricular response; AEC, absolute eosinophilic count; ANCA, anti-neutrophil cytoplasmic antibodies; LVEF, left ventricular ejection fraction; LGE, late gadolinium enhancement; CMR, cardiac magnetic resonance; NEM, necrotizing eosinophilic myocarditis; EM, eosinophilic myocarditis; SEndo, subendocardial; SEpi, subepicardial; ICTh, intracardiac thrombosis; Tm, transmural; GCs, glucocorticoids; p, pulse; AZA, azathioprine; CYC, cyclophosphamide; MTX, methotrexate; RTX, rituximab; MPZ, mepolizumab, IVIG, intravenous immunoglobulin; VF, ventricular fibrillation; na, not available.*

*Revised 2021 Five-Factor Score (FFS): The presence of each factor is given one point. The FFS score is converted to 0 if none of the factors are present, 1 if one factor is present, and 2 if two or more factors are present, respectively.

Herein, we describe a previously undiagnosed case of probable EGPA with the development of acute EM, which was successfully treated with corticosteroid treatment. This case provides three clinical suggestions.

First, the clinical course, in this case, was acute EM without obvious vasculitis, which led to a delayed diagnosis of probable EGPA.

The typical symptoms of systemic vasculitis in EGPA include constitutional symptoms (fever, malaise, and weight loss), myalgia, mono/polyneuropathy (numbness, tingling, muscle weakness, and pain), skin symptoms (purpura and non-pruritic nodules), and gastrointestinal ischemic symptoms such as abdominal pain. However, none of the above symptoms, except for low-grade fever, were observed in our case. The present case exemplifies the following three diagnostic challenges of EGPA.

First, HSM was suspected based on the medication history. External factors such as exposure to allergens, infection, or vaccination can induce the development of EGPA. However, the symptoms precipitated even after discontinuation of the suspected drugs, and the histological findings on EMB confirmed that HSM was less likely. Anti-asthmatic drugs, such as leukotriene receptor antagonists and anti-IgE antibodies can trigger the development of EGPA; however, a direct causal relationship in our case was unclear. The anti-asthmatic effects of the drugs might have delayed the systemic administration of corticosteroids, resulting in the manifestation of EGPA (47).

Second, the main histopathological findings of EGPA, other than eosinophilic infiltration, were not observed in our case. EMBs in patients with EGPA-associated EM often show EM without vasculitis (2), which is probably attributed to the possibility of heterogeneous distribution of vasculitis, the small number of EMB samples, and the limited biopsy sites including the left ventricular apex and interventricular septum (Table 1). Besides, in the autopsy cases, it was very likely to detect evidence of vasculitis because the whole heart was able to be analyzed. Therefore, EGPA-associated EM cannot be ruled out based on the lack of histologic evidence of vasculitis in EMBs. In addition, because the histopathologic findings may vary by the phase of the disease, our patient might have been during the early vasculitic phase of the disease. Furthermore, the cases of EGPA-associated EM have a high frequency of coexisting asthma (94.7%) (Table 1). Therefore, inhaled corticosteroids for asthma, and corticosteroid prophylaxis for allergic reactions to the contrast media, might have contributed to the absence of typical histopathologic findings in our patient. Subsequently, this patient presented with a mononeuritis multiplex which was significantly associated with systemic vasculitis (48), leading us to believe that this patient was diagnosed with probable EGPA.

A third diagnostic challenge is the potential clinical and biological overlap between EGPA and idiopathic HES. HES is a sporadic disorder that is diagnosed based on the following criteria: elevated AEC (>1,500 cells/μL on at least two occasions), and/or pathologic confirmation of tissue hypereosinophilia. With great advances in the knowledge of eosinophil biology and molecular diagnostics, the classification of HES subgroups is evolving rapidly. The following six variants of HES have been proposed (49): (i) myeloid HES, (ii) lymphocytic HES, (iii) overlap HES—eosinophilic disorders overlapping in presentation with idiopathic HES (e.g., EGPA), (iv) associated HES (eg, parasite infections, drug hypersensitivities, or immunodeficiency), (v) familial HES, and (vi) idiopathic HES—eosinophilic disorders of unknown etiology. Because AECs are high (average: 6,716; range: 1,850–30,609) and ANCAs are often undetectable (87%) in EGPA-associated EM, the clinical characteristics of EGPA-associated EM are similar to those of idiopathic HES (50). Thus, it is crucial to distinguish between the two because they differ in treatment and prognosis. EGPA and idiopathic HES can be differentiated based on vasculitis (clinical or histological). Our case review showed that patients with established EGPA-associated EM had a high prevalence of neurological involvement (52.6%, Table 1). Considering ANCA-negativity and the absence of significant vasculitis, our case was difficult to distinguish from idiopathic HES. Generally, asthma is not mandatory for the diagnosis of EGPA (3) and can also be found in patients with any variants of HES. Nevertheless, given that the overwhelming majority of patients with EGPA-associated EM have concomitant asthma of 94.7% as shown in Table 1, our case underscores the critical importance of preferentially considering EGPA as the causative etiology in cases of EM with asthma. A study analyzing 179 cases with histologically proven EM reported the prevalence of a history of asthma to be 68% in the EGPA group, 21% in the idiopathic/undefined group, and 23% in the HES group (51), which suggested that EGPA might not have been diagnosed due to the absence of obvious vasculitis in the latter two groups.

As a second clinical suggestion, both CMR and EMB were valuable for the final diagnosis of probable EGPA in our case.

CMR enables the characterization of myocardial tissue properties (inflammation, thrombus, and fibrosis) and yields high diagnostic performance in identifying acute myocarditis (81% sensitivity, 71% specificity, and 79% accuracy) (52). Typical CMR findings of EGPA-associated EM include subendocardial LGE in the apical and mid-left ventricles. In our case, the following unique CMR findings led to a strong suspicion of EGPA-associated EM. CMR revealed diffuse patchy subendocardial defects on FPP at rest, which were superimposed on abnormal lesions on LGE and T2-weighted images. Taken together, these observations were suggestive of microvascular dysfunction caused by acute inflammation, which was consistent with previous reports (53). Similar findings were observed in a patient with cardiac syndrome X, which is characterized by unexplained chest pain (54). Although coronary luminal stenosis, thrombus, or spasms have been proposed as possible causes of angina symptoms in patients with EGPA, the ACS-like symptoms reported in our case can be explained by microvascular dysfunction. Notably, in our case, multiple foci on the initial LGE were resolved following the corticosteroid treatment. Similar foci have been reported in some cases of EGPA-associated EM, and are presumed to be specific to vasculitis, which can be an indicator for EGPA-associated EM (35, 43, 55). Further studies on the relationship between foci on LGE and their respective pathologies are warranted. In addition, EMB provides useful information on the nature and distribution of inflammatory infiltrates. EGPA and HSM can be differentiated based on the histopathological findings. The histological features of EGPA include tissue eosinophilia, extravascular eosinophilic granuloma, and necrotizing vasculitis. However, the latter two are rarely seen in cardiac pathology (56) (Table 1), whereas HSM is characterized by interstitial prominent eosinophilic infiltrates without myocardial necrosis or fibrosis (57). The distinct pattern of endo/peri-myocardial eosinophilic infiltration and degranulation, accompanied by the presence of myocardial necrosis and fibrosis in EMB, was a crucial finding for the definitive diagnosis of acute EM and differentiating it from HSM. EMB is the golden standard for histological diagnosis of myocarditis, but it is an invasive method with limitations such as serious procedure-related complications or sampling errors. With an excellent diagnostic accuracy of CMR in acute myocarditis due to great advances in imaging technology, the usefulness of non-invasive CMR alone in diagnosing acute myocarditis has been widely reported (52, 58, 59). Therefore, a single approach of either CMR or EMB is now considered sufficient for the diagnosis of acute myocarditis. However, each approach has its advantages and disadvantages in cases of acute EM that develops as the first manifestation of EGPA without clinical vasculitis as per our case. Therefore, our case underscores that the combination of CMR and EMB provides diagnostic synergy in the exploratory diagnosis of EGPA in patients with suspected acute EM.

Our final clinical suggestion is that timely corticosteroid treatment allowed significant recovery and normalization of the cardiac structure and function in our case.

The primary treatment for EGPA is systemic glucocorticoids. Additional immunosuppressive agents should be considered in patients with progressive, refractory, or relapsing diseases. Despite administering multiple immunomodulators, many cases of EGPA-associated EM were directly linked to fatal cardiac complications and incomplete recovery of cardiac function (Table 1). In our case, systemic and oral corticosteroids resulted in complete recovery and normalization of the cardiac structure and function within about 2 months. Considering the malignant features of EGPA-associated EM, our case underscores the significance of early recognition and treatment of this disease.

## Conclusion

We present a case of probable EGPA with subclinical mononeuritis multiplex that developed acute EM and was successfully treated with systemic corticosteroid therapy. The EGPA-associated EM is a rare yet potentially life-threatening disorder that can be cured if treated appropriately and timely. However, without obvious vasculitis, accurate diagnosis of EGPA-associated EM is challenging. Therefore, clinicians should be aware of this rare disease and consider using both CMR and EMB diagnostic approaches for patients with suspected EM.

## Data Availability Statement

The original contributions presented in the study are included in the article/Supplementary Materials, further inquiries can be directed to the corresponding author/s.

## Ethics Statement

Authorization for the use of case information and materials was obtained from the Institutional Review Board of Narita-Tomisato Tokushukai Hospital. The authors confirm that written consent for the submission and publication of this case report, including the images and associated movie, was obtained from the patient.

## Author Contributions

HY contributed in creating the clinical design and concept, interpreted the data, drafted, and revised the manuscript. HY, KH, and TH acquired the clinical data. JI performed the CMR analyses. YI performed the pathological analyses. All authors discussed and approved the manuscript and authorized its submission for publication.

## Conflict of Interest

The authors declare that the research was conducted in the absence of any commercial or financial relationships that could be construed as a potential conflict of interest.

## Publisher's Note

All claims expressed in this article are solely those of the authors and do not necessarily represent those of their affiliated organizations, or those of the publisher, the editors and the reviewers. Any product that may be evaluated in this article, or claim that may be made by its manufacturer, is not guaranteed or endorsed by the publisher.

## Supplementary Material

Supplementary Figure 1 ECG at admission **(A)** and on day 21 **(B)**. An initial ECG reveals evidence of pathologic Q waves in V2 to V3, and a significant left axis deviation at −57°, which is consistent with a left anterior fascicular block. Notably, the follow-up ECG after treatment of corticosteroid shows the resolution of all abnormal findings recognized during the initial ECG. ECG, electrocardiogram.

Supplementary Figure 2 Clinical effects of the corticosteroid treatment on TTE. Follow-up TTE on day 56 after the corticosteroid therapy reveals a full recovery of the ventricular function and GLS values of the LV (LVDd, 49 mm; LVEF, 62%; GLS −16.1%, respectively) **(A–C)**. Ao, aorta; GLS, global longitudinal strain; LA, left atrium; LV, left ventricle; LVDd, left ventricular end-diastolic diameter; LVEF, left ventricular ejection fraction; TTE, transthoracic echocardiography.

Supplementary Figure 3 A timeline for the case presentation. EGPA, eosinophilic granulomatosis with polyangiitis; EM, eosinophilic myocarditis; HES, hypereosinophilic syndrome; LGE, late gadolinium enhancement; LV, left ventricular.

Supplementary Video 1 Pre-treatment transthoracic echocardiography. Transthoracic echocardiography at the initial presentation in a parasternal short-axis view reveals mild diffuse thickening of the left ventricular wall and decreased cavity size with a reduced ejection fraction of 43%.

Supplementary Video 2 Pre-treatment myocardial first-pass perfusion deficits on CMR. Short-axis CMR imaging demonstrates that myocardial first-pass perfusion at rest shows patchy and circumferential subendocardial perfusion deficits. CMR: cardiac magnetic resonance.

Supplementary Video 3 Post-treatment transthoracic echocardiography on day 21. Post-treatment follow-up transthoracic echocardiography in a parasternal short-axis view reveals significant resolution of the left ventricular wall thickening with concomitant improvement in cavity size and ventricular function with an ejection fraction of 50%.

Supplementary Video 4 Post-treatment transthoracic echocardiography on day 56. Note the fully recovered cardiac function observed on echocardiography.

Supplementary Video 5 Changes in myocardial first-pass perfusion deficits on CMR following corticosteroid treatment. Note the significant improvement in the abnormal first-pass perfusion deficits at admission on post-treatment follow-up CMR. CMR, cardiac magnetic resonance.

## Abbreviations

EGPA: eosinophilic granulomatosis with polyangiitis
EM: eosinophilic myocarditis
ECG: electrocardiogram
AEC: absolute eosinophilic count
ANCA: anti-neutrophil cytoplasmic antibodies
LV: left ventricular
ACS: acute coronary syndrome
CMR: cardiac magnetic resonance
FPP: first-pass perfusion
LGE: late gadolinium enhancement
HES: hypereosinophilic syndrome
HSM: hypersensitivity myocarditis
EMB: endomyocardial biopsy
ACR: American College of Rheumatology.
