# Supplementary figures and images for: The Diagnostic Challenge of Eosinophilic Granulomatosis With Polyangiitis Presenting as Acute Eosinophilic Myocarditis: Case Report and Literature Review
Source: Front Cardiovasc Med. 2022 Jul 7;9:913724. doi: 10.3389/fcvm.2022.913724 (PMC9300862; doi:10.3389/fcvm.2022.913724)

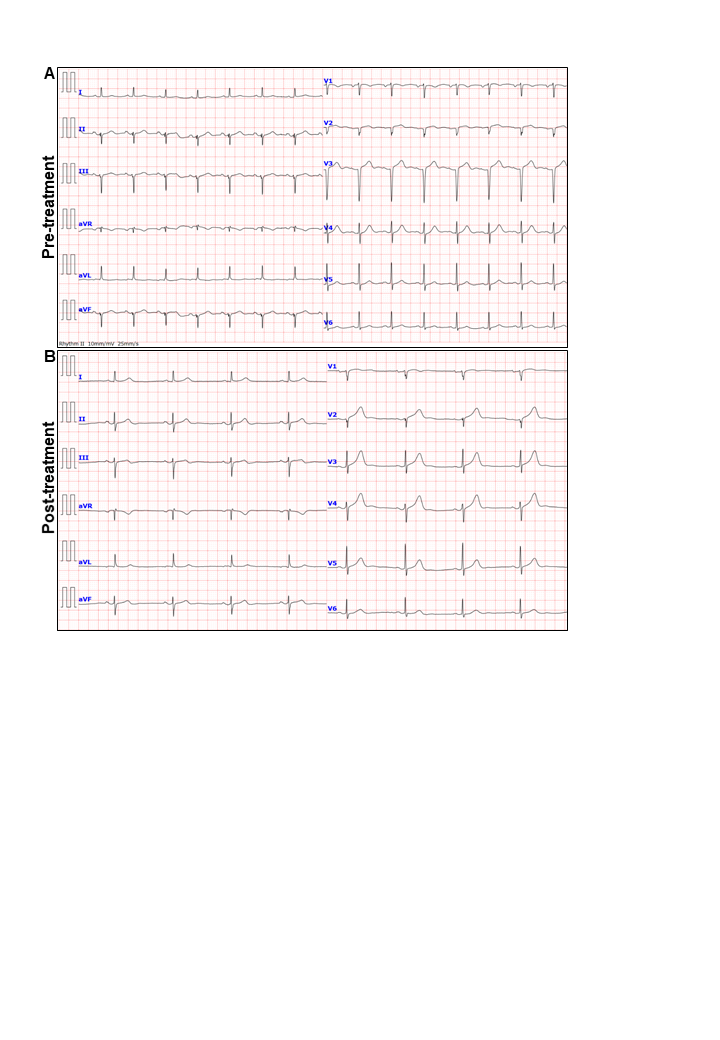

Supplement: Supplementary Figure 1 — ECG at admission (A) and on day 21 (B). An initial ECG reveals evidence of pathologic Q waves in V2 to V3, and a significant left axis deviation at −57°, which is consistent with a left anterior fascicular block. Notably, the follow-up ECG after treatment of corticosteroid shows the resolution of all abnormal findings recognized during the initial ECG. ECG, electrocardiogram. [file Image_1.TIF]

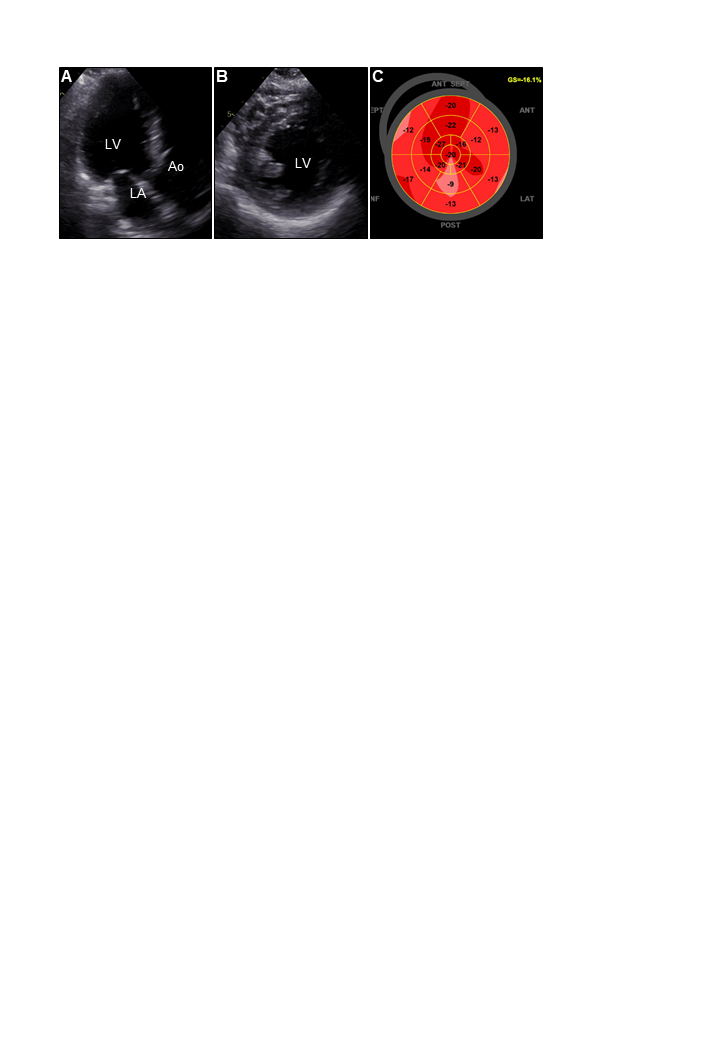

Supplement: Supplementary Figure 2 — Clinical effects of the corticosteroid treatment on TTE. Follow-up TTE on day 56 after the corticosteroid therapy reveals a full recovery of the ventricular function and GLS values of the LV (LVDd, 49 mm; LVEF, 62%; GLS −16.1%, respectively) (A–C). Ao, aorta; GLS, global longitudinal strain; LA, left atrium; LV, left ventricle; LVDd, left ventricular end-diastolic diameter; LVEF, left ventricular ejection fraction; TTE, transthoracic echocardiography. [file Image_2.TIF]

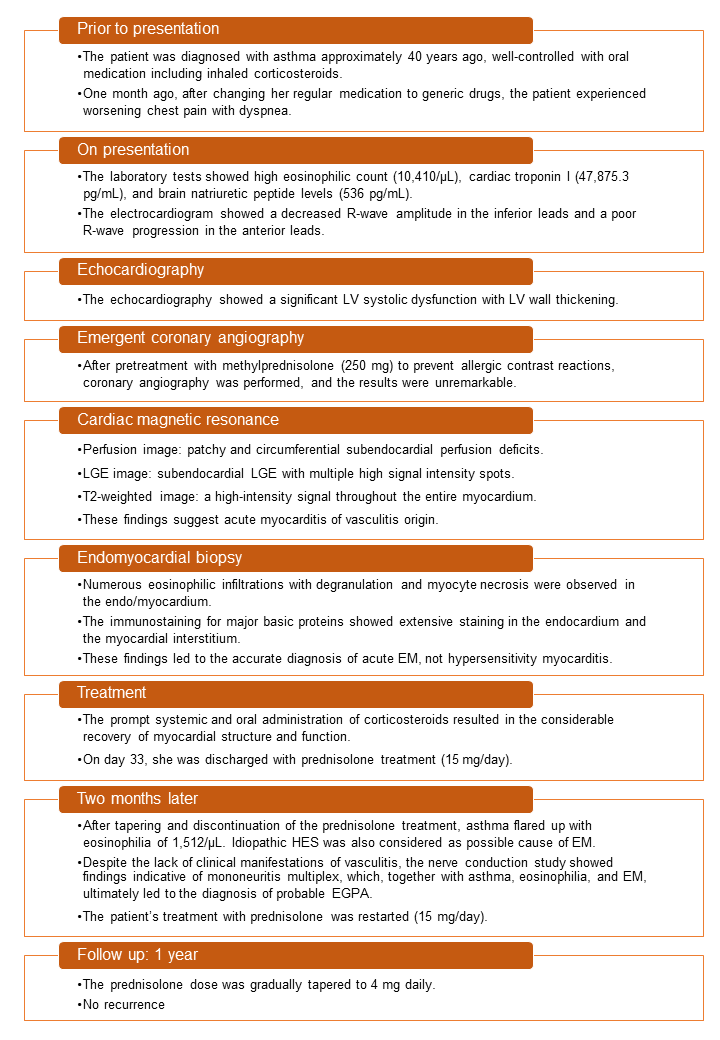

Supplement: Supplementary Figure 3 — A timeline for the case presentation. EGPA, eosinophilic granulomatosis with polyangiitis; EM, eosinophilic myocarditis; HES, hypereosinophilic syndrome; LGE, late gadolinium enhancement; LV, left ventricular. [file Image_3.TIF]
